# The Atlanta Medical Society

**Published:** 1882-06

**Authors:** 


### THE ATLANTA MEDICAL SOCIETY.

*Reported for the Atlanta Medical Register.*

ATLANTA, April, 1882.

The President, Dr. W. S. Armstrong, presiding.

Dr. G. G. Roy detailed the history of two cases which simulated pregnancy in the array of symptoms presented, and which were calculated to mislead and confuse even the most cautious and most careful observer, as subsequent developments proved that pregnancy did not exist in either case.

Dr. Jno. Thad. Johnson reported a case occurring in his practice which presented nothing remarkable, but would prove valuable, he thought, as a contribution to the history of an interesting subject.

He removed from a young man 17 years of age, by the lateral operation of lithotomy, a stone measuring one inch in its long diameter by three-quarters of an inch transversely. After removal it was accidentally dropped and broken into two fragments, disclosing in the center a bean which had served as the nucleus.

The boy, when confronted with this witness, confessed that three and a half years previously, while toying with his penis, he had inserted the bean, and being ashamed to acknowledge the act, he had suffered in silence and never admitted the rash performance until the moment of his detection.

Of course, all kinds of neuclei are found imbedded in vesical calculi, and the literature of the subject is filled with curious illustrations of this sort.

Dr. James B. Baird reported several cases of chorea, some of them presenting unusual features, and asked the views of members as to the most satisfactory treatment of this affection.

Among the suggestions offered in response to this inquiry Dr. G. G. Roy urged the value of the cold salt shower-bath, administered by preference about eleven o'clock in the morning, when the stomach is empty and the activity of the nervous centres is at its height. It should be followed by active friction. He regards the cold bath as a powerful sedative.

Dr. F. H. O'Brien referred to a case of membranous croup. The mother of the child discovered the membrane upon the fauces. The most prominent symptoms were loss of voice and cough. As the result of emetics administered, the membrane was expelled. The aphonia continued for three weeks. The little patient recovered under the use of tonics, etc. The child was a blonde. Fair children are said to be more liable to the disease than others. Since recovery an impediment in speech remains. Advised the faradic current to the larynx. This advice was not taken, and the trouble has not abated.

Dr. James B. Baird alluded, in connection with this case, to his experience with faradic electricity in similar affections. Spoke of an infant in whom the uvula was perfectly flaccid, vibrating with every action of respiration, and producing a loud, snorting sound, extremely disagreeable to hear. Removal of a portion of the uvula failed to cause amendment. Relief was ultimately obtained by the use of faradism to the muscles of the palate.

Dr. W. S. Armstrong related a case to show the effect of treatment. About one year ago was called to see a child aged two months, very much emaciated, could not sleep, had difficulty of breathing, and what the people call the "snuffles." While nursing it had to stop for every breath. It was the seventh child of its mother, who was aged only twenty-three years. All the other children had died when about two weeks old. The parents were extremely anxious to rear this one. He was satisfied that syphilis caused all the trouble. Ordered bichloride of mercury and iodide of potassium—one one-hundredth of a grain of the former and two grains of the latter at every dose. In two weeks the worst symptoms were relieved, and in a surprisingly short time the child was fat and rosy. One week ago saw the child again. The treatment had long ago been discontinued. He had lost flesh; had an eruption upon the body; and his teeth showed signs of the disease. The treatment was resumed, and the improvement has been remarkable.

The father has acknowledged to once having had syphilis.

Dr. Jno. Thad. Johnson said he thought it the best practice not to act upon suspicion in commencing antisyphilitic treatment, but considers it the safest plan to await unmistakable evidences of the existence of the disease before launching out upon a systematic course of treatment. The mercurial treatment should not be undertaken until the presence of the disease is confirmed.

Dr. F. H. O'Brien inquired if Dr. J. was afraid that he would mask the symptoms by the treatment, or that the remedies would prove injurious?

Dr. Johnson replied that he believed in being certain as to the diagnosis, and after the treatment is commenced pushing it unremittingly. It should not be started until the necessity for it obtained, but then it should be faithfully and persistently used.

Dr. Armstrong observed if doubt as to the syphilitic nature of a particular case existed he should not hesitate to make a test by employing proper remedies suited to that condition; a few days are usually quite sufficient to clear up any uncertainty.

Dr. E. L. Connally referred to the case of a widow lady whom he treated a long time for some uterine disease. He finally observed suspicious symptoms in one of her children six years old. He afterwards learned that her husband had had some syphilitic manifestations. Mercury and iodide of potassium promptly relieved the trouble; the leucorrhœa ceased and the tenderness and enlargement of the ovaries speedily vanished.

Dr. Jno. Thad. Johnson recited the history of two cases presenting an unusually protracted period of incubation. Two young men had intercourse with the same woman about the same time. Chancre developed in one on the sixty-seventh day and in the other on the sixty-ninth day. This was the first sign of the disease. The sores were not overlooked, but were discovered as soon as they appeared. Is certain that no deception was practiced by either of the gentlemen.

